# A Unique Case of Pancreatic Mass due to Pancreatic Elastofibromatosis

**DOI:** 10.1155/2016/2697187

**Published:** 2016-11-06

**Authors:** Abhinav Goyal, Deepanshu Jain, Ishfaq Bhat, Shailender Singh

**Affiliations:** ^1^Einstein Medical Center, Philadelphia, PA, USA; ^2^University of Nebraska Medical Center, Omaha, NE, USA

## Abstract

Elastofibroma is a benign tumor of the musculoskeletal system characterized by the abnormal accumulation of elastinophilic fibers. It has been classically described for subscapular region but has been reported in several musculoskeletal sites over the years and rarely even in the GI tract but never in pancreas. We therefore present the case of a 45-year-old female who presented with intermittent abdominal pain. CT of abdomen revealed 1.4 cm pancreatic neck lesion without peripancreatic lymphadenopathy. Endoscopic ultrasound (EUS) guided FNA was nondiagnostic. Surgical resection was performed with central pancreatectomy. Histopathology revealed well demarcated nodules of hypocellular collagen with abundant elastic fibers, characteristic of pancreatic elastofibroma. Treatment is not needed unless symptomatic and surgical resection is the preferred therapeutic option when indicated. This case adds another entity to the differential diagnosis of pancreatic mass lesions.

## 1. Introduction

Elastofibroma is a benign slow growing soft tissue tumor composed of characteristic elastinophilic fibers. According to WHO classification, it is currently considered to be a member of benign fibroblastic or myofibroblastic soft tissue tumors found in the periscapular region [1]. It was initially described as a subscapular connective tissue tumor but, over the years, has been seen to involve several musculoskeletal sites like greater trochanter, ischial tuberosity, and deltoid [2–4]. It has been rarely reported in the GI tract, usually involving the stomach and sigmoid colon but to this date has never been reported in pancreas [5, 6]. We therefore present the first case of elastofibroma in pancreas.

## 2. Case Report

**Figure 1:**
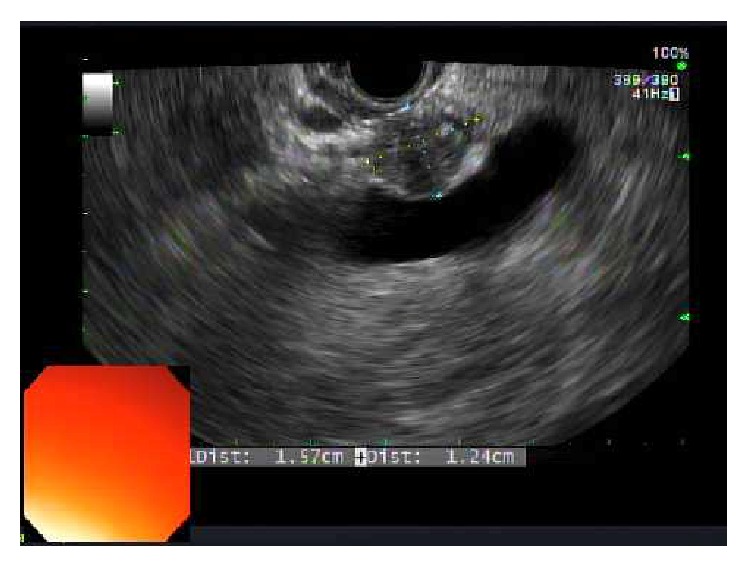
EUS image of the pancreatic elastofibroma.

**Figure 2:**
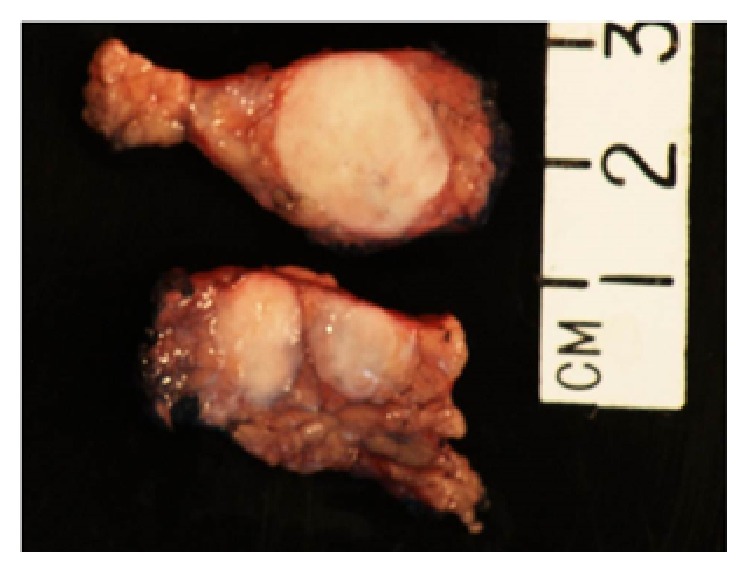
Gross specimen of the resected pancreas with the mass lesion (pancreatic elastofibroma).

**Figure 3:**
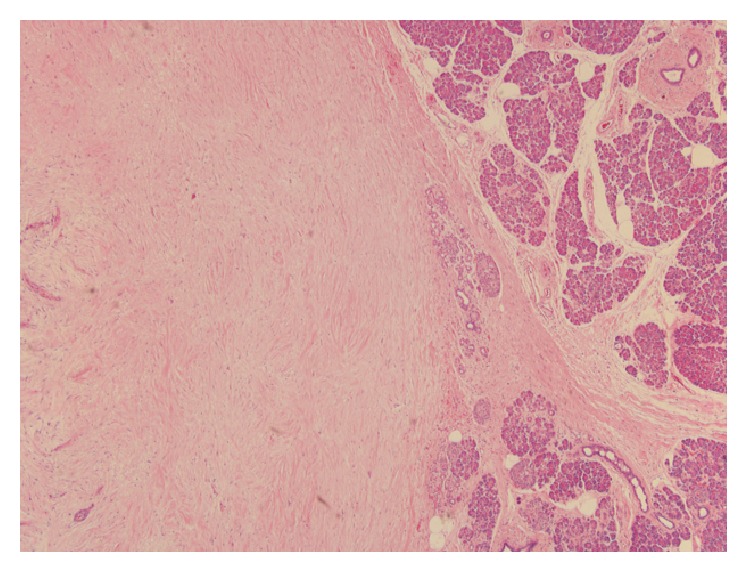
Histology of the resected tumor at a magnification of 40x showing abundance of elastic fibers.

**Figure 4:**
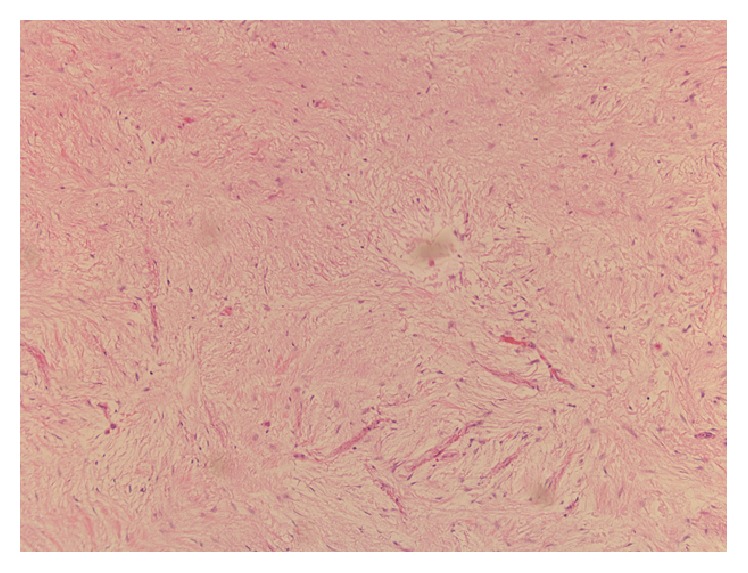
Histology of the resected tumor at a magnification of 100x showing abundant elastic fibers.

A 45-year-old female with history of joint pains (HLA B27 positive, seronegative inflammatory arthritis) presented with intermittent abdominal pain of few years' duration. General physical exam and serum chemistries were unremarkable. CT scan of her abdomen revealed a 1.4 by 1.4 cm hypodense lesion in the neck of pancreas just anterior to portal confluence, with no vascular involvement. Another 6 mm nodule thought to represent either a lobulation of the larger mass or a new lesion was also seen adjacent to it. No peripancreatic lymphadenopathy was apparent. Subsequently, an endoscopic ultrasound (EUS) was done. It showed a 2.5 × 1.2 cm hypoechoic mass in the pancreatic neck region (Figure 1). FNA of the pancreatic mass was nondiagnostic and was repeated. Repeat EUS-FNA was reported negative for malignancy. But, due to the concerning appearance of the lesion it was decided to proceed with the surgical resection of the mass. Therefore, a central pancreatectomy was performed and the specimen (Figure 2) was sent for histopathological analysis. Histopathology revealed three well demarcated nodules of hypocellular collagen with abundant elastic fibers (highlighted by Verhoeff's Van Gieson or VVG stain) and admixed bland spindle to stellate cells, without necrosis or mitotic activity (Figures 3 and 4). Immunostaining of spindle cells was negative for epithelial membrane antigen (EMA), H-caldesmon, pankeratin, CD117, S100, desmin, and beta-catenin nuclear stain. Hence, a diagnosis of pancreatic elastofibroma was made. During the subsequent follow-up of about 2.5 years till date, she has had recurrent acute pancreatitis of unknown etiology, involving both the head and tail region on imaging studies. Option of total pancreatectomy with islet cell autotransplantation is being considered as definitive future management.

## 3. Discussion

Elastofibroma is a relatively uncommon benign tumor of the musculoskeletal system. Elastofibroma has a predilection for subscapular area and is more commonly found in elderly females. It is characterized by abnormal accumulation of elastin fibers, pathogenesis of which is unclear [7]. Several theories have been put forward to explain the pathogenesis of thoracoscapular elastofibroma including mechanical friction driven degeneration of collagen fibers and reactive hyperplasia of the fibroelastic cells secondary to an unknown stimulus [8, 9].

Over the years it has been reported at several locations throughout the musculoskeletal system including foot, spine, deltoid, greater trochanter, chest wall, and face [2, 3, 10–12]. It is very rare to find this tumor at visceral sites. There are only a few case reports of elastofibroma involving the GI tract, specifically stomach, colon, and small intestines, but to our knowledge it has never been reported in pancreas [5, 6, 13, 14]. This case therefore adds another entity to the differential diagnosis of pancreatic mass lesions.

Despite the uncertain pathogenesis, treatment is usually not recommended unless it is symptomatic. Most of the literature is in the form of case reports but surgical resection is the preferred therapeutic option when definitive treatment is required [7, 10, 15, 16].
